# Selenoprotein P, peroxiredoxin-5, renalase, and total antioxidant status in patients with suspected obstructive sleep apnea

**DOI:** 10.1007/s11325-023-02880-7

**Published:** 2023-07-26

**Authors:** Karolina Czerwińska, Lidia Januszewska, Iwona Markiewicz-Górka, Aleksandra Jaremków, Helena Martynowicz, Krystyna Pawlas, Grzegorz Mazur, Rafał Poręba, Paweł Gać

**Affiliations:** 1 Division of Environmental Health and Occupational Medicine, Department of Population Health, Wroclaw Medical University, Mikulicza-Radeckiego 7, 50-368 Wroclaw, PL Poland; 2 Department of Internal and Occupational Diseases, Hypertension and Clinical Oncology, Wroclaw Medical University, Borowska 213, 50-556 Wroclaw, PL Poland

**Keywords:** Antioxidants, Hypertension, Obstructive sleep apnea, Peroxiredoxin-5, Renalase, Selenoprotein P

## Abstract

**Purpose:**

The aim of this study was to investigate the relationship between selenoprotein P, peroxiredoxin-5, renalase, total antioxidant status (TAS), mean blood pressure (mBP), and apnea-hypopnea index (AHI).

**Methods:**

The study group consisted of 112 patients hospitalized to verify the diagnosis of obstructive sleep apnea (OSA). The inclusion criteria were consent to participate in the study and age ≥ 18 years. Patients with active proliferative disease, severe systemic diseases, or mental diseases were excluded from the study. Each patient underwent full polysomnography and had blood pressure measured. Blood samples were collected and laboratory test was performed.

**Results:**

Among 112 patients enrolled, there was a statistically significant negative linear correlation between blood pressure values (sBP, dBP, mBP) and selenoprotein P, renalase, and TAS levels. Similarly, there was a negative linear correlation between AHI and selenoprotein P, renalase, and TAS levels, but none between AHI and peroxiredoxin-5. Based on the obtained regression models, higher selenoprotein P, peroxiredoxin-5, and renalase levels were independently associated with higher TAS. Lower mBP values were independently associated with the use of antihypertensive drugs, higher TAS, and younger age. Male gender, higher BMI, and higher mBP were independently associated with higher AHI.

**Conclusions:**

Higher concentrations of selenoprotein P, peroxiredoxin-5, and renalase were associated with higher TAS, which confirms their antioxidant properties. There was an indirect connection between tested antioxidants and blood pressure values.

## Introduction

Redox imbalance plays a role in the pathogenesis of cardiovascular diseases (CVDs). Overwhelmed cellular antioxidant capacity is described in the course of such diseases as hypertension, atherosclerosis, or ischemic heart disease [1, 2]. These diseases remain the leading cause of death worldwide, and any factors that could be used in their prevention, diagnosis, or therapy are in-demand [3]. There is potential in using antioxidants as a supplement for treating or preventing cardiovascular diseases. While many substances with antioxidant properties have been researched, none has been recommended for this purpose thus far [4]. It is related to a large number of conflicting reports [5, 6]. It seems that such supplementation has potential; however, the difficulty lies in choosing the dose and substance suitable for patients with a specific disease or risk factor [7]. Therefore, the search for appropriate antioxidants continues. In our study, we focused on peroxiredoxin-5, which is a known antioxidant, as well as on selenoprotein P and renalase, which are suspected to exert antioxidant properties.

SelP is a unique protein that contains multiple selenocysteine (Sec) residues [8]. It is primarily produced in the liver and released into the bloodstream after the removal of its signal peptide. The abbreviation “P” in SelP signifies its presence in the plasma. SelP consists of two domains: the N-terminal and C-terminal domains. The N-terminal Sec residue acts as the enzyme’s active site, reducing phospholipid hydroperoxide. Meanwhile, the nine C-terminal Sec residue serves as the Se transporter [8]. In vitro studies have shown that SelP acts as a phospholipid hydroperoxide glutathione peroxidase and a peroxynitrite reductase [9]. Steinbrenner et al. reported that SelP derived from human plasma safeguards low-density lipoproteins (LDL) from oxidation [9]. Moreover, it is suggested that cells pretreated with SelP are protected from oxidative damage caused by tert-butyl hydroperoxide due to an increase in the production of intracellular selenoenzymes [10]. It appears that SelP plays a role in promoting cardiovascular health by working together with endothelial cells. Plasma SelP not only supplies selenium to these cells, but also acts as an external antioxidant. This helps safeguard plasma proteins against damage from peroxynitrite-induced oxidation and nitration [10]. In the case of selenium deficiency, the concentration of plasma SelP decreases, making it a possible indicator of selenium’s nutritional status [9, 10]. However, more research is needed to fully understand SelP’s role in maintaining redox balance and cardiovascular health.

Renalase was first described in 2005 and since then it became an object of scientific interest due to its proposed ability to catalyze circulating neurotransmitters and its promising antihypertensive effects [11]. Based on preliminary animal model reports, it was found that administering renalase intravenously can lower blood pressure, heart rate, and cardiac contractility [11]. This effect was entitled to renalase breaking down catecholamines in the bloodstream. However, subsequent research on its enzymatic activity did not support these initial findings. Instead, it was discovered that renalase helps to oxidize isomeric forms of β-NAD(P)H and recycle them by forming β-NAD(P)+ [12]. These isomers were found to inhibit some β-NAD(P)H-dependent enzymes in vitro, namely, porcine heart lactate dehydrogenase (LDH) and *Escherichia coli* malate dehydrogenase (MDH) [12]. There is speculation that some human enzymes which use β-NAD(P)H as a cofactor might be inhibited by β-NAD(P)H isoforms too. From this standpoint, renalase seems to act as a scavenger enzyme that protects cells against the accumulation of substances that may negatively affect other enzymatic reactions. The data on renalase’s involvement in redox balance in humans is scarce. We have discussed recent findings on renalase’s enzymatic and non-enzymatic activity in our previous work [13].

Peroxiredoxins (Prdxs) are a family of peroxidases that participate in maintaining thiol balance by reducing organic hydroperoxides, H_2_O_2_, and peroxynitrite [14]. According to scientific research, Prdxs have the potential to be used in the diagnosis and treatment of cardiovascular disease (CVD) [15–18]. Developing derivatives or mimicking the catalytic activity of Prdxs could be a promising approach for antioxidant therapy in treating CVD [18]. It is important to note that the majority of research focuses on Prdx-1. Prdx-5 is the most recently discovered member of the peroxiredoxins family, and its impact on CVD requires further investigation.

Fighting ROS and keeping the redox balance require the cooperation of many compounds with antioxidant properties. The total antioxidant capacity of the body is composed of both endogenous and food-derived antioxidants. The collaboration among different antioxidants offers superior protection against reactive oxygen or nitrogen species. Therefore, the total antioxidant status is a more useful measure of biological information than the measurement of individual components, as it considers the combined effect of all antioxidants present in the plasma.

Obstructive sleep apnea (OSA) is a respiratory sleep disorder caused by repeated obstruction of the upper airway during sleep, leading to interruptions in oxygen flow, arousal, and fragmented sleep [19, 20]. The severity of OSA is measured by the apnea/hypopnea index (AHI), which counts the number of respiratory events per hour. A diagnosis of OSA is made when the AHI is five or more. Mild OSA is indicated by an AHI from 5 up to 15; moderate OSA is indicated by an AHI from 15 up to 30, and severe OSA requires an AHI of 30 or more [19]. It is important to note that OSA increases the risk of cardiovascular disease [21]. Repeated episodes of apnea cause sympathetic over-activation and acute inflammation, leading to an increase in cardiovascular morbidity and mortality [22].

The aim of this study was to investigate the relationship between selenoprotein P, peroxiredoxin-5, renalase, total antioxidant status (TAS), mean blood pressure (mBP), and apnea-hypopnea index (AHI).

## Materials and methods

The study group included patients who were admitted consecutively to an internal medicine clinic for the purpose of verifying the diagnosis of obstructive sleep apnea. Group size was determined using a sample size calculator. The selection conditions were as follows: population size 2.9 million (population size of the macroregion from which patients are referred to our study center), fraction size 0.5, maximum error 10%, confidence level 95%. The required minimum number of subjects was 96. To be eligible for the study, participants had to be 18 years or older and provide their consent. Patients with severe systemic diseases, severe mental illness/disorders, or active proliferative disease were excluded from the study.

A single-night recording of PSG was conducted at the Sleep Laboratory of the Department of Internal Medicine, Occupational Diseases, Hypertension and Clinical Oncology, Wroclaw Medical University, Poland. The Nox-A1 machine from Nox Medical, Iceland, was used according to the standard diagnostic criteria for nocturnal recording. The assessment of the polysomnograms was carried out in 30-s epochs based on the American Academy of Sleep Medicine (AASM) 2013 criteria for sleep scoring, by a qualified physician (H.M.) from the Sleep Laboratory. For a thorough explanation of PSG methodology, please refer to our recent work [23].

Blood was collected from the patient’s ulna vein in the morning after polysomnography. The blood samples were stored at a constant temperature until renalase determinations were performed simultaneously on all samples. The serum renalase levels were checked using the E3109Hu kit ELISA from the Bioassay Technology Laboratory in Shanghai, China, as per the manufacturer’s instructions. The renalase concentration was measured in ng/ml, with a reference range of 1–400 ng/ml. The ELISA test used had a sensitivity of 0.52 ng/ml, and the coefficient of intra- and inter-assay variation was less than 8% and 10%, respectively.

We utilized the E1809h ELISA Kit for Human SeP from ElAab in Wuhan, China, to measure serum selenoprotein P. Following the manufacturer’s instructions diligently, we expressed the selenoprotein P concentration in ng/ml. The coefficient of intra-assay variation was less than 4.9% and inter-assay variation was less than 7.1%.

We used the E0703h ELISA Kit for Human Peroxiredoxin-5, mitochondrial (ElAab) from Wuhan, China, to measure serum peroxiredoxin-5. The test was conducted following the manufacturer’s instructions, and the results were presented as nanograms/milliliter (ng/ml). The reference range of the assay was 0.78–50 ng/ml.

Because the effects of different antioxidants are additive, the total antioxidant status (TAS) was used to measure the overall antioxidant status of the body. The USA Antioxidant Assay Kit No709001 was used to measure TAS in our study group. This protocol does not differentiate between aqueous- and lipid-soluble antioxidants. Instead, it evaluates the overall antioxidant activity of all constituents such as vitamins, proteins, lipids, glutathione, and uric acid. This assay measures the ability of antioxidants in the sample to inhibit the oxidation of ABTS® (2,2′-Azino-di-[3-ethylbenzthiazoline sulfonate]) to ABTS®^•+^ by metmyoglobin. The capacity of the antioxidants in the sample to prevent ABTS® oxidation is compared with that of Trolox, a water-soluble tocopherol analog, and is quantified as millimolar Trolox equivalents.

The auscultatory method using mercury sphygmomanometer was used to measure blood pressure. During the measurement, the patients were relaxed and seated comfortably with their feet resting on the ground. The arm was relaxed, uncovered, and supported at the level of the heart. Blood pressure was measured twice by the Korotkoff method. Mean blood pressure (mBP) was calculated using the formula: mean blood pressure = diastolic blood pressure + 1/3*(systolic blood pressure − diastolic blood pressure). Arterial hypertension was diagnosed when the mean of two measurements was at least 140 mmHg for systolic pressure or 90 mmHg for diastolic pressure. In a situation where the patient declared taking any antihypertensive drugs, arterial hypertension was diagnosed, regardless of the measured blood pressure values.

We used Dell Statistica 13 software (Dell Inc., USA) to conduct statistical analyses. Mean and standard deviation were used to express quantitative variables, while percentages were used for qualitative variables. We tested the distribution of variables using the W-Shapiro-Wilk test. For normally distributed quantitative variables, we used the *T*-test for further statistical analysis. To analyze non-normally distributed quantitative variables, we used the Mann-Whitney *U* test. For qualitative variables, we used the chi-square test of maximum likelihood. Additionally, correlation and regression analyses were conducted to establish the relationship between the variables. The Pearson correlation *r* factor was used for normal distribution, while the Spearman *r* factor was used for non-normal distribution. We used multivariable stepwise backward regression to identify possible predictor variables for changes in mBP, number of AHI events, and TAS level. Variables were removed from the model based on *p* values.

Ethical approval for this study was obtained from the Bioethics Committee of Wroclaw Medical University. Before the study, all participants gave their informed consent.

ClinicalTrials.gov Identifier: NCT05040516.

## Results

The study group comprised 112 participants, 52.7% men (*n* = 59) and 47.3% women (*n* = 53). The complete characteristics of the study group are presented in Table 1.

**Table 1:** Characteristics of the study group (*n* = 112)

	Mean	Standard deviation
Age (years)	49.8	14.7
BMI (kg/m^2^)	28.7	5.3
sBP (mmHg)	139.5	20.7
dBP (mmHg)	89.6	12.8
mBP (mmHg)	106.2	14.7
AHI (events/h)	18.0	18.8
Average SpO2 (%)	93.2	2.5
Minimum SpO2 (%)	83.5	8.0
SpO2 <90% (%)	9.5	18.23
Selenoprotein P (ng/ml)	1.49	1.88
Peroxiredoxin-5 (ng/ml)	1.82	4.52
Renalase (ng/ml)	186.98	213.86
TAS (nM)	1.15	0.33
	Number	Percent
Men	59	53
Women	53	47
Overweight/obesity	55	49
HTN	43	38
Diuretics	19	17
β-Blockers	22	20
ACE inhibitors	20	18
Angiotensin receptor blockers	13	12
Calcium channel blockers	12	11
OSA	80	71
Mild OSA	32	29
Moderate OSA	21	189
Severe OSA	27	24
Type 2 diabetes	10	9
Coronary artery disease	8	7

*AHI* apnea-hypopnea index, *BMI* body mass index, *dBP* diastolic blood pressure, *HTN* arterial hypertension, *mBP* mean blood pressure, *OSA* obstructive sleep apnea, *sBP* systolic blood pressure, *SpO2* oxygen saturation; *TAS* total antioxidant status

For detailed analysis, the patients were divided into subgroups. First, four subgroups were distinguished based on the diagnosis of HTN and OSA. Subgroup A consisted of patients diagnosed with both HTN and OSA (HTN+, OSA+), subgroup B of patients with HTN but without OSA (HTN+, OSA−), subgroup C of patients diagnosed with OSA but without HTN (HTN−, OSA+), and subgroup D of patients without HTN and OSA (HTN−, OSA−). The second division included median values of mBP (Me = 105 mmHg) and AHI (Me = 10.95). The following subgroups were distinguished: E—patients with high mBP and high AHI (mBP ≥ Me, AHI ≥ Me), F—patients with high mBP but low AHI (mBP ≥ Me, AHI<Me), G—patients with low mBP and high AHI (mBP < Me, AHI ≥ Me), and H—patients with low mBP and low AHI (mBP < Me, AHI < Me).

Statistical analysis of the selenoprotein P, peroxiredoxin-5, and renalase levels, as well as total antioxidant status in the study subgroups, are presented in Table 2. Patients in the subgroup designated as D (HTN− OSA−) had statistically significantly higher selenoprotein P and TAS levels when compared to subgroup A (HTN+ OSA+). There were no significant differences between the other subgroups in terms of these parameters. Peroxiredoxin-5 and renalase levels did not differ significantly between any of the subgroups A–D.

**Table 2:** Selenoprotein P, peroxiredoxin-5, renalase, and total antioxidant status in the study subgroups

Subgroup	Selenoprotein P (ng/ml)	Peroxiredoxin-5 (ng/ml)	Renalase (ng/ml)	TAS (nM)
A (*n* = 38)	1.04 ± 0.96	1.09 ± 0.99	132.28 ± 189.87	1.07 ± 0.26
B (*n* = 5)	0.52 ± 0.16	2.56 ± 3.09	308.10 ± 266.10	1.15 ± 0.10
C (*n* = 42)	1.78 ± 2.46	2.34 ± 5.81	203.34 ± 207.20	1.15 ± 0.41
D (*n* = 27)	1.96 ± 1.88	1.85 ± 5.38	216.69 ± 238.15	1.29 ± 0.31
*p*	A vs. D: <0.05	ns	ns	A vs. D: <0.05
HTN+ (*n* = 43)	0.98 ± 0.92	1.27 ± 1.43	152.72 ± 204.34	1.08 ± 0.25
HTN− (*n* = 69)	1.85 ± 2.25	2.16 ± 5.62	208.64 ± 218.36	1.20 ± 0.37
*p*	<0.05	ns	ns	<0.05
OSA+ (*n* = 80)	1.42 ± 1.92	1.76 ± 4.34	169.16 ± 200.98	1.11 ± 0.34
OSA− (*n* = 32)	1.71 ± 1.79	1.97 ± 5.03	230.97 ± 240.50	1.26 ± 0.29
*p*	ns	ns	ns	<0.05
E (*n* = 40)	1.02 ± 1.19	1.07 ± 1.03	136.67 ± 192.75	1.05 ± 0.25
F (*n* = 18)	1.47 ± 1.67	3.10 ± 6.41	187.87 ± 216.22	1.20 ± 0.35
G (*n* = 16)	1.79 ± 2.82	3.30 ± 9.13	216.91 ± 192.17	1.29 ± 0.51
H (*n* = 38)	1.94 ± 2.09	1.87 ± 1.63	225.59 ± 238.28	1.19 ± 0.29
*p*	E vs. H: <0.05	ns	E vs. H: <0.05	E vs. G: <0.05 E vs. H: <0.05
mBP ≥ Me (*n* = 58)	1.16 ± 1.36	1.72 ± 3.79	152.84 ± 199.95	1.10 ± 0.29
mBP < Me (*n* = 54)	1.89 ± 2.30	1.93 ± 5.23	223.02 ± 223.84	1.22 ± 0.37
*p*	<0.05	ns	<0.05	<0.05
AHI ≥ Me (*n* = 56)	1.23 ± 1.79	2.03 ± 5.15	160.01 ± 194.31	1.12 ± 0.36
AHI < Me (*n* = 56)	1.78 ± 1.96	1.62 ± 3.82	213.46 ± 230.13	1.19 ± 0.31
*p*	ns	ns	ns	ns

*AHI* apnea-hypopnea index, *HTN* arterial hypertension, *mBP* mean blood pressure, *Me* median value, *OSA* obstructive sleep apnea, *TAS* total antioxidant status

Dividing patients by diagnosis of arterial hypertension showed significant differences in selenoprotein P and TAS levels between HTN+ (*n* = 43) and HTN− (*n* = 69) subgroups, but no differences in peroxiredoxin-5 and renalase levels. Division by diagnosis of obstructive sleep apnea found statistical differences in TAS levels between OSA+ (*n* = 80) and OSA− (*n* = 32) patients, but not for the other parameters.

Statistically significant differences for selenoprotein P, renalase, and TAS levels, but no for peroxiredoxin level, were found when dividing patients by median values of mBP and AHI. Subjects in the E subgroup (high mBP, high AHI) had lower selenoprotein P, renalase, and TAS levels when compared to subjects in the H subgroup (low mBP, low AHI). Moreover, there was a statistically significant difference between TAS levels between the E subgroup (high mBP, high AHI) and the G subgroup (low mBP, high AHI).

Selenoprotein P, renalase, and TAS levels, but no peroxiredoxin level, were statistically significantly lower in patients with high mBP (≥Me, *n* = 58) when compared to patients with low mBP (<Me, *n* = 54). No such differences were found when comparing patients with high AHI (≥Me, *n* = 56) and low AHI (<Me, *n* = 56).

Statistically significant positive linear correlation was observed between TAS and selenoprotein P (*r* = 0.57), peroxiredoxin-5 (*r* = 0.48), and renalase (*r* = 0.25) levels. No significant linear relationship was found between selenoprotein P, peroxiredoxin-5, and renalase levels.

There was a statistically significant negative linear correlation between blood pressure values (sBP, dBP, mBP) and selenoprotein P, renalase, and TAS levels. No such correlation was found for the peroxiredoxin-5 level. Similarly, there was a negative linear correlation between AHI and selenoprotein P, renalase, and TAS levels, but none between AHI and peroxiredoxin-5. In addition, it has been shown that there are positive linear correlations between mean saturation and TAS, and minimum saturation and TAS. The results of the linear correlation analysis including the correlation coefficient are presented in Table 3.

**Table 3:**
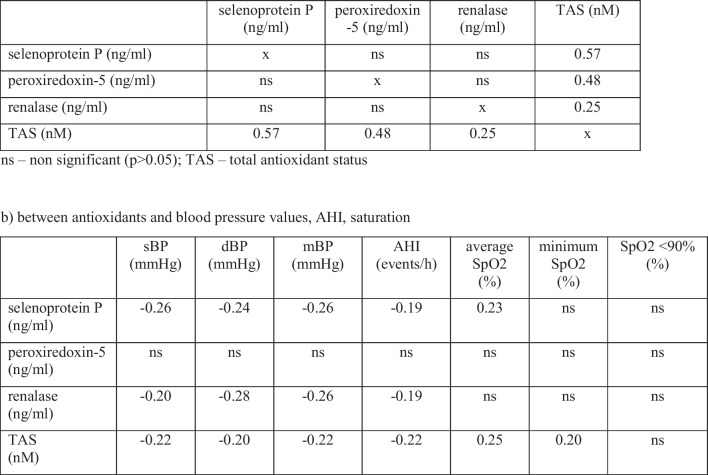
The results of the correlation analysis in the study group

*AHI* apnea-hypopnea index, *dBP* diastolic blood pressure, *mBP* mean blood pressure, *ns* non-significant (*p* > 0.05), *sBP* systolic blood pressure, *SpO2* oxygen saturation, *TAS* total antioxidant status

**Fig. 1:**
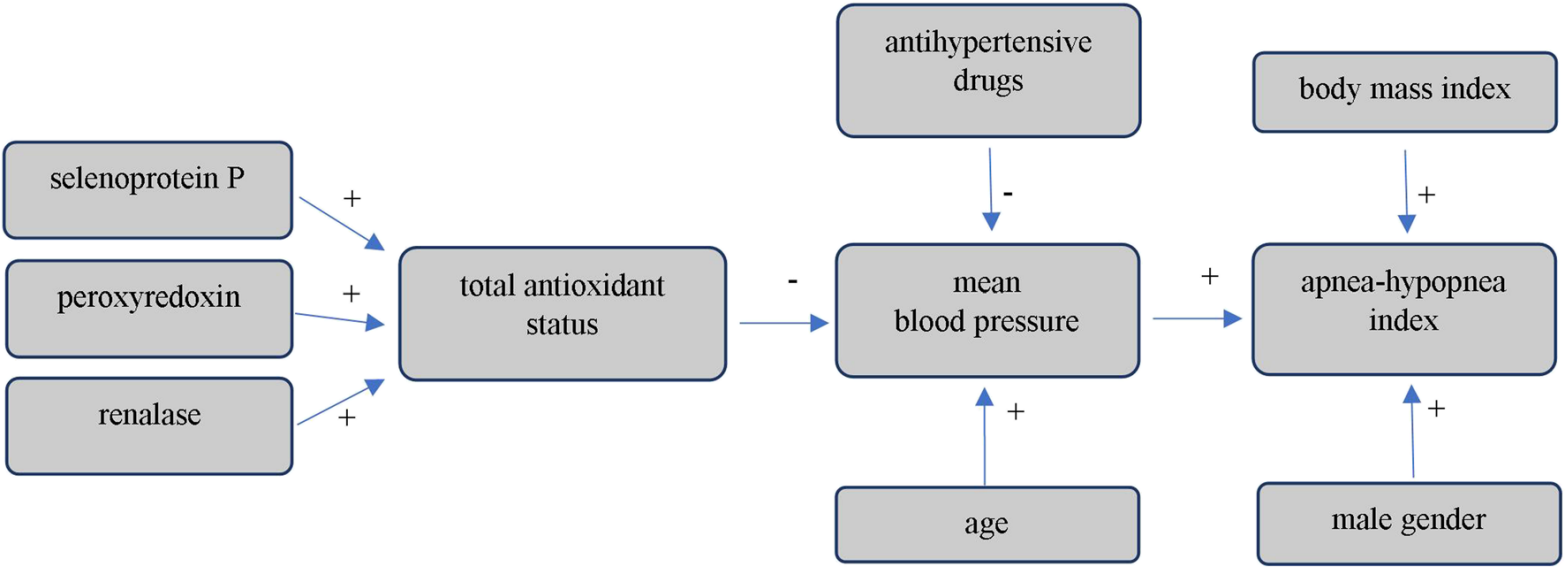
Diagram summarizing the regression analysis

A multivariable stepwise backward regression analysis was performed for three different dependent variables: mBP, AHI, and TAS level. A final model obtained for mBP as a dependent variable was as follows: mBP = 94.645 − 10.761 antihypertensive drugs − 6.486 TAS + 0.24 age. Based on the obtained regression model, use of antihypertensive drugs, higher TAS, and younger age were independently associated with lower mBP values. A final model obtained for AHI as a dependent variable was as follows: AHI = −54.449 + 11.540 male gender + 0.660 BMI + 0.621 mBP. Based on the obtained regression model, it was shown that male gender, higher BMI, and higher mBP values were independently associated with higher AHI values. A final model obtained for TAS level was as follows: TAS = 0.889 + 0.103 selenoprotein P + 0.040 peroxiredoxin-5 + 0.001 renalase. Based on the obtained regression model, higher selenoprotein P, peroxiredoxin-5, and renalase levels were independently associated with higher TAS. Detailed results of multivariable stepwise backward regression analysis in the study group are presented in Table 4. Figure 1 shows a diagram summarizing the results of regression analysis.

**Table 4:**
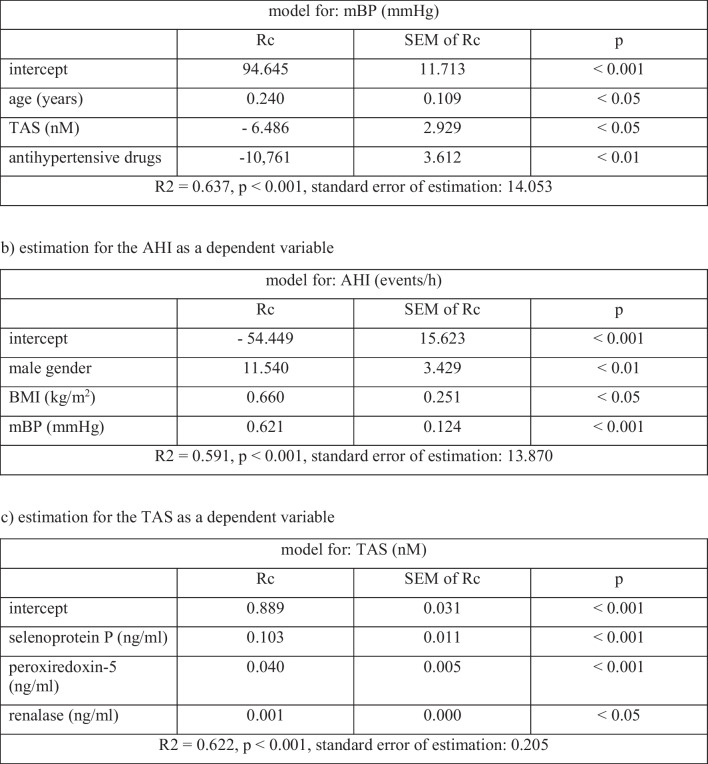
Results of multivariable stepwise backward regression analysis in the study group

*AHI* apnea-hypopnea index, *BMI* body mass index, *mBP* mean blood pressure, *Rc* regression coefficient, *SEM* standard error of mean, *TAS* total antioxidant status

## Discussion

The most important result of this study is the finding of a relationship between the tested laboratory parameters, mBP, and AHI. Higher concentrations of selenoprotein P, peroxiredoxin-5, and renalase were associated with higher TAS. Higher TAS was associated with lower mBP values, and lower mBP values were associated with lower AHI index. These results indicate an indirect connection between selenoprotein P, peroxiredoxin-5, and renalase levels and blood pressure values. It is an argument for the concept that a decrease in antioxidant levels, redox imbalance, and intensification of oxidative stress provoke an increase in blood pressure values. This causative role of oxidative stress in hypertension pathophysiology has also been proposed by other authors [24–26]. Increased intracellular ROS levels are described to promote oxidative modification of signaling proteins and cause altered vascular signaling. Moreover, ROS were found to increase vasoconstriction and reduce endothelium-dependent vasodilation by increasing the production of prostanoids [27]. The concept that overwhelmed cellular antioxidant capacity promotes hypertension is also supported by findings that blood pressure can be lowered by antioxidants, ROS scavengers, and Nox inhibitors [26, 28]. Nevertheless, this matter seems more complicated as there are studies with contrary results [29, 30].

It is worth noting that renalase has only recently been proposed as a scavenger enzyme [12]. Initially, it was thought to oxidize circulating catecholamines but that assumption was not proven in more detailed trials [11, 31]. Instead, Beaupre et al. recognized 2- and 6-dihydroNAD(P) molecules to be the real substrates for renalase [12]. These molecules are isomeric forms of native β-NAD(P)H (4-dihydroNAD(P)H) that arise either by nonspecific reduction of β-NAD(P)+ or by tautomerization of β-NAD(P)H. Renalase serves to oxidase these isomers and recycles them by forming β-NAD(P)+. This action seems highly favorable for the cell because β-NAD(P)+ isomeric forms were found to competitively bind to β-NAD(P)+ dependent enzymes and inhibit their activity [12]. In short, renalase is a scavenger enzyme that protects cells against the accumulation of substances that may negatively affect other enzymatic reactions. However, these assumptions are not based on human studies and it is yet to be established whether human enzymes are prone to inhibition by isomeric forms of β-NAD(P)H. We have discussed enzymatic and non-enzymatic activity of renalase in our previous work [13]. In this study, we have found a positive linear relationship between renalase blood concentration and TAS level which seems to confirm renalases’ antioxidant properties. To our knowledge, this is the first study to describe a direct connection between renalase level and total antioxidant status. However, the strength of this correlation was weaker than for selenoprotein P or peroxiredoxin-5. The mechanism behind renalases’ antioxidant activity remains unknown. It may be connected with the fact that many enzymes involved in redox balance, such as a family of nonphagocytic NADPH oxidases or nitric oxide synthase, are NAD(P)+ dependent. However, to date, no studies have addressed this issue. Another possible mechanism involves renalase’s extracellular action and its impact on calcium balance, which is considered an important part of maintaining redox homeostasis [32]. Recently plasma membrane calcium ATPase isoform (PMCA4b) was found to serve as a receptor for extracellular renalase [33]. The interaction between renalase and PMCA4b receptor is involved in maintaining optimal Ca^2+^ homeostasis. Alterations in extracellular calcium levels influence intracellular calcium levels and possibly play an important role in the pathogenesis of essential hypertension [34]. Nevertheless, the clinical relevance of the interaction between renalase and PMCA4b receptor remains undiscovered.

In our study, we have found that during subgroup analyses selenoprotein P was the substance that differed the most between the study subgroups, especially in the context of blood pressure. It was statistically significantly higher in subgroup D (HTN− OSA−) than in subgroup A (HTN+ OSA+). It was also significantly higher in subgroup H (low mBP, low AHI) than in subgroup E (high mBP, high AHI). The differences were also significant when dividing patients by the diagnosis of hypertension or mBP only (Table 3). Therefore, we suggest that among studied substances, the disturbances in selenoprotein P concentration are the most involved in lowering TAS in this group of patients. We further suggest that increasing selenoprotein P concentration may be a promising therapeutic strategy to increase TAS and lower blood pressure. The possible involvement of selenoprotein P in the maintenance of cardiovascular health was also described by Schomburg et al. In their study, quintiles of selenoprotein P concentration were related to the risk of all-cause mortality, cardiovascular mortality, and a first cardiovascular event. The main conclusion was that 20% with the lowest selenoprotein P concentration had markedly increased risk of cardiovascular morbidity and mortality [35]. However, this was a population-based prospective cohort study. Clinical trials testing if cardiovascular morbidity and mortality can be reduced in subjects belonging to this low selenoprotein P group are still needed.

It is worth noting that even though statistically significant positive linear correlation was observed between TAS and peroxiredoxin-5, we did not find any significant differences in its levels between chosen subgroups. It may suggest that not all antioxidants are of equal importance in this patient group.

Results of multivariable stepwise backward regression analysis showed that AHI was directly influenced by BMI, male gender, and mBP but not TAS. We did not find significant differences between selenoprotein P, peroxiredoxin-5, renalase, and TAS levels when dividing patients by the diagnosis of OSA or the number of AHI. In short, AHI was not directly influenced by TAS or antioxidants. However, estimation for the mBP as a dependent variable found a relationship between mBP values and AHI, which may indicate an indirect connection between TAS and AHI through mBP. An increase in TAS may decrease mBP values and consequently decrease AHI. Our results stay consistent with the notion that weight reduction and proper blood pressure control are one of the most effective methods to reduce the number of apnea/hypopnea episodes [36]. Our recent study showed an inversely proportional linear relationship between renalase concentration and AHI values in the entire study group, suggesting the association between OSA and renalase [20]. In this study, we further investigated this issue. We did not find significant differences between renalase levels when dividing patients by the diagnosis of OSA, or by the AHI value. However, renalase level was significantly lower in patients with mBP ≥ Me than in patients with mBP < Me. It seems that the relationship between renalase and OSA is indirect and that renalase impacts AHI through its influence on TAS and mBP.

The strengths of our research include the performance of a full polysomnographic examination in patients with clinical suspicion of obstructive sleep apnea, the inclusion of other polysomnographic examination parameters (saturation) in the analyses apart from the AHI, the performance of polysomnographic examinations in a recognized sleep laboratory, the analysis of all polysomnographic examinations by one qualified physician with extensive clinical and scientific experience, standardization of blood pressure measurement, determination of the total antioxidant status in addition to the determination of selected antioxidants, and the performance of multivariate analyses taking into account the impact of potential modifying factors on the examined relationships.

There are limitations to this study. The number of subjects involved in the study was rather small. In addition, the lack of randomization must also be considered a significant limitation. The group of patients consisted of successive patients admitted to the hospital. In the characteristics of the group, the relatively high value of the saturation index and the high percentage of patients using several antihypertensive drugs are noteworthy. The limitations of the research methodology include the lack of night-time blood pressure monitoring, as well as the subjective selection of antioxidants.

## Conclusions

In our study, higher concentrations of selenoprotein P, peroxiredoxin-5, and renalase were associated with higher TAS, which confirms their antioxidant properties. The strongest correlation was found for selenoprotein P, which was also the substance that differed the most between the study subgroups, especially in the context of blood pressure. In contrast, peroxiredoxin-5 level correlated with TAS but did not differ significantly between study subgroups, which may suggest that not all antioxidants are of equal importance in this patient group.

## Data Availability

Data will be made available on reasonable request.

## References

[1] Madamanchi NR, Runge MS (2013). Redox signaling in cardiovascular health and disease. Free Radic Biol Med.

[2] Moris D et al (2017) The role of reactive oxygen species in the pathophysiology of cardiovascular diseases and the clinical significance of myocardial redox. Ann Transl Med 5(16). 10.21037/atm.2017.06.27 10.21037/atm.2017.06.27 PMC5566734 28861423

[3] Virani SS (2021). Heart disease and stroke statistics - 2021 update: a report from the American Heart Association. Circulation.

[4] Visseren FLJ (2021). 2021 ESC guidelines on cardiovascular disease prevention in clinical practice. Eur Heart J.

[5] Jenkins DJA (2018). Supplemental vitamins and minerals for CVD prevention and treatment. J Am Coll Cardiol.

[6] Brigo F, Storti M, Lochner P, Tezzon F, Nardone R (2014) Selenium supplementation for primary prevention of cardiovascular disease: proof of no effectiveness. Nutr Metab Cardiovasc Dis 24(1). 10.1016/j.numecd.2013.08.013 10.1016/j.numecd.2013.08.013 24418382

[7] Cammisotto V (2021). The role of antioxidants supplementation in clinical practice: focus on cardiovascular risk factors. Antioxidants.

[8] Burk RF, Hill KE (2009). Selenoprotein P-expression, functions, and roles in mammals. Biochim Biophys Acta Gen Subj.

[9] Steinbrenner H, Speckmann B, Klotz LO (2016). Selenoproteins: antioxidant selenoenzymes and beyond. Arch Biochem Biophys.

[10] Steinbrenner H (2006). Selenoprotein P protects endothelial cells from oxidative damage by stimulation of glutathione peroxidase expression and activity. Free Radic Res.

[11] Xu J (2005). Renalase is a novel, soluble monoamine oxidase that regulates cardiac function and blood pressure. J Clin Investig.

[12] Beaupre BA, Hoag MR, Roman J, Försterling FH, Moran GR (2015). Metabolic function for human renalase: oxidation of isomeric forms of β-NAD(P)H that are inhibitory to primary metabolism. Biochemistry.

[13] Czerwińska K, Poręba R, Gać P (2022). Renalase—a new understanding of its enzymatic and non-enzymatic activity and its implications for future research. Clin Exp Pharmacol Physiol.

[14] Rhee SG, Woo HA, Kil IS, Bae SH (2012). Peroxiredoxin functions as a peroxidase and a regulator and sensor of local peroxides. J Biol Chem.

[15] Tang C et al (2020) Peroxiredoxin-1 ameliorates pressure overload-induced cardiac hypertrophy and fibrosis. Biomed Pharmacother 129. 10.1016/j.biopha.2020.110357 10.1016/j.biopha.2020.110357 32531679

[16] Jiang L et al (2020) Peroxiredoxin-1 overexpression attenuates doxorubicin-induced cardiotoxicity by inhibiting oxidative stress and cardiomyocyte apoptosis. Oxid Med Cell Longev 2020. 10.1155/2020/2405135 10.1155/2020/2405135 PMC7411498 32802259

[17] Kisucka J (2008). Peroxiredoxin1 prevents excessive endothelial activation and early atherosclerosis. Circ Res.

[18] Jeong SJ, Park JG, Oh GT (2021) Peroxiredoxins as potential targets for cardiovascular disease. Antioxidants 10(8). 10.3390/antiox10081244 10.3390/antiox10081244 PMC8389283 34439492

[19] Jordan AS, McSharry DG, Malhotra A (2014). Adult obstructive sleep apnoea. The Lancet.

[20] Martynowicz H (2021). Renalase and hypertension-demographic and clinical correlates in obstructive sleep apnea. Sleep Breath.

[21] Benjamin EJ (2019). Heart disease and stroke statistics-2019 update: a report from the American Heart Association. Circulation.

[22] Eisele HJ, Markart P, Schulz R (2015) Obstructive sleep apnea, oxidative stress, and cardiovascular disease: evidence from human studies. Oxid Med Cell Longev 2015. 10.1155/2015/608438 10.1155/2015/608438 PMC4475750 26167241

[23] Czerwińska K, Januszewska L, Markiewicz-Górka I, Jaremków A, Martynowicz H, Pawlas K, Mazur G, Poręba R, Gać P (2023). Selenoprotein P, peroxiredoxin-5, renalase and selected cardiovascular consequences tested in ambulatory blood pressure monitoring and echocardiography. Antioxidants.

[24] Rodrigo R, González J, Paoletto F (2011). The role of oxidative stress in the pathophysiology of hypertension. Hypertens Res.

[25] Massaro M, Scoditti E, Carluccio MA, de Caterina R (2019). Oxidative stress and vascular stiffness in hypertension: a renewed interest for antioxidant therapies?. Vascul Pharmacol.

[26] Touyz RM, Rios FJ, Alves-Lopes R, Neves KB, Camargo LL, Montezano AC (2020). Oxidative stress: a unifying paradigm in hypertension. Can J Cardiol.

[27] Montezano AC, Dulak-Lis M, Tsiropoulou S, Harvey A, Briones AM, Touyz RM (2015). Oxidative stress and human hypertension: vascular mechanisms, biomarkers, and novel therapies. Can J Cardiol.

[28] Drummond GR, Selemidis S, Griendling KK, Sobey CG (2011). Combating oxidative stress in vascular disease: NADPH oxidases as therapeutic targets. Nat Rev Drug Discov.

[29] Vivekananthan DP, Penn MS, Sapp SK, Hsu A, Topol EJ (2003). Use of antioxidant vitamins for the prevention of cardiovascular disease: meta-analysis of randomised trials. The Lancet.

[30] Baradaran A, Nasri H, Rafi Eian-Kopaei M (2014) Oxidative stress and hypertension: possibility of hypertension therapy with antioxidants. J Res Med Sci 358 PMC4115353 25097610

[31] Beaupre BA, Hoag MR, Moran GR (2015). Renalase does not catalyze the oxidation of catecholamines. Arch Biochem Biophys.

[32] Matuz-Mares D, González-Andrade M, Araiza-Villanueva MG, Vilchis-Landeros MM, Vázquez-Meza H (2022) Mitochondrial calcium: effects of its imbalance in disease. Antioxidants 11(5). 10.3390/antiox11050801 10.3390/antiox11050801 PMC9138001 35624667

[33] Wang L, Velazquez H, Chang J, Safirstein R, Desir GV (Apr. 2015) Identification of a receptor for extracellular renalase. PLoS One 10(4). 10.1371/journal.pone.0122932 10.1371/journal.pone.0122932 PMC4407985 25906147

[34] Behradmanesh S, Nasri H (2013). Association of serum calcium with level of blood pressure in type 2 diabetic patients. J Nephropathol.

[35] Schomburg L, Orho-Melander M, Struck J, Bergmann A, Melander O (2019) Seleno protein-P deficiency predicts cardiovascular disease and death. Nutrients 11(8). 10.3390/nu11081852 10.3390/nu11081852 PMC6723215 31404994

[36] Sun HHB, Sun S, Sun HHB, Sun S (2020). Diagnosis and management of obstructive sleep apnea. Surgical management of head and neck pathologies.

